# Examiner seniority and experience are associated with bias when scoring communication, but not examination, skills in objective structured clinical examinations in Australia

**DOI:** 10.3352/jeehp.2018.15.17

**Published:** 2018-07-18

**Authors:** Lauren Chong, Silas Taylor, Matthew Haywood, Barbara-Ann Adelstein, Boaz Shulruf

**Affiliations:** 1 Clinical Skills Teaching Unit, Prince of Wales Hospital, Sydney, Australia; 2 University of New South Wales, Sydney, Australia; 3 Prince of Wales Clinical School, University of New South Wales, Sydney, Australia; 4 Office of Medical Education, University of New South Wales, Sydney, Australia; 5 Centre for Medical and Health Sciences Education, University of Auckland, Auckland, New Zealand; Hallym University, Korea

**Keywords:** Examiner, Bias, Communication, Examination, Objective structured clinical examination, Australia

## Abstract

**Purpose:**

The biases that may influence objective structured clinical examination (OSCE) scoring are well understood, and recent research has attempted to establish the magnitude of their impact. However, the influence of examiner experience, clinical seniority, and occupation on communication and physical examination scores in OSCEs has not yet been clearly established.

**Methods:**

We compared the mean scores awarded for generic and clinical communication and physical examination skills in 2 undergraduate medicine OSCEs in relation to examiner characteristics (gender, examining experience, occupation, seniority, and speciality). The statistical significance of the differences was calculated using the 2-tailed independent t-test and analysis of variance.

**Results:**

Five hundred and seventeen students were examined by 237 examiners at the University of New South Wales in 2014 and 2016. Examiner gender, occupation (academic, clinician, or clinical tutor), and job type (specialist or generalist) did not significantly impact scores. Junior doctors gave consistently higher scores than senior doctors in all domains, and this difference was statistically significant for generic and clinical communication scores. Examiner experience was significantly inversely correlated with generic communication scores.

**Conclusion:**

We suggest that the assessment of examination skills may be less susceptible to bias because this process is fairly prescriptive, affording greater scoring objectivity. We recommend training to define the marking criteria, teaching curriculum, and expected level of performance in communication skills to reduce bias in OSCE assessment.

## Introduction

The sources of bias that may influence objective structured clinical examination (OSCE) scores are well understood, and include the halo [1], ‘hawk-dove’ [2], contrast [3], and site [4] effects, in addition to the examiner’s familiarity with the students [1] and the duration of the examining period [1]. Recently, research has attempted to establish the magnitude of the impact of these various sources of bias and their propensity to influence particular domains of assessed competency, such as communication. For example, a previous study attributed up to 11% of variance in awarded marks to the examiner contrast effect in OSCE-type settings [3]. With respect to gender bias, male examiners have been found to award significantly higher communication skills ratings to female candidates [2], although this may be confounded by females’ tendency to perform better in this context than their male counterparts. While the effects of different biases within the communication skills domain have been explored in some depth [5], the same does not hold true for specific assessed competencies, such as physical examination or practical skills.

Attempts have also been made to delineate the somewhat inter-related effects of examining experience, the clinical seniority of the examiner, and examiner occupation on OSCE ratings. It has been recognised that examiners become more stringent within a single session as they examine more candidates; however, greater leniency is exhibited by untrained examiners than by trained ones [6]. It can be argued that ‘background’ and ‘experience’ are conflated in some studies, in much the same way that medical student examiners marking more generously than both ‘teaching doctors’ or ‘senior academic(s)’ may be explained in terms of their clinical and examining inexperience [7]. However, these 2 variables are not necessarily correlated, and the general failure in the literature to accurately distinguish among examiner characteristics with respect to experience, occupation, and rank/seniority makes it almost impossible to draw any inferences regarding the relative importance of these variables.

With regard to examiner occupation, it is important to understand that this descriptor encompasses not only clinicians who may or may not specialise in the assessment subject, but also doctors-by-degree who work full-time in academia. While some evidence suggests that physician examiners’ familiarity with a speciality does not influence the marks they award, examiners may use themselves as a reference point when grading a student, leading to harsher candidate ratings as they become more experienced [3].

The present paper therefore aimed to provide a clear account of the biases associated with examining experience, examiner occupation, and clinical seniority of the examiner with respect to communication and physical examination domain scores in an undergraduate OSCE. We individually analysed the relative influences of these often-conflated examiner characteristics in the context of specific competency domains. These findings will contribute to a greater understanding of the sources and impact of examiner bias, thus enabling the targeted implementation of strategies that ensure the continued validity of the OSCE as an assessment tool.

## Methods

### Ethical statement

The data used in the present study were derived from the OSCEs administered to our 2014 and 2016 year 2 medicine student cohorts. These 2 assessments were identical in their composition. Ethical approval was granted by the University of New South Wales (UNSW) Human Research Ethics Committee (Ref: HC15421), and the study was exempted from the requirement to obtain informed consent from the subjects.

### Criteria and data

Our OSCE focused on 3 domains graded across 9 criteria (items): generic communication skills (4 items); clinical communication skills (i.e., medical history taking; 3 items); and physical examination skills (2 items). A grade was awarded for each criterion and post-assessment processing assigned a numerical mark to each grade, as follows: fail (F= 3/10); borderline pass (P-= 5/10); clear pass (P= 7/10); and exceeded expectations/distinction (P+= 9/10). The numerical marks were totalled to give an overall score for each student within each domain. Grades were entered into an in-house app presented on iPads to the examiners. Every student was assessed by a single examiner per station, producing 1 mark for each of the 9 criteria. In total, there were 6 stations per candidate (total number of items= 54).

Data on examiner characteristics were collected at each OSCE sitting, and included gender, examining experience, occupation, seniority, and speciality. Experience was defined based on the number of times the examiner had evaluated medical student OSCEs at UNSW prior to the present study, and was categorised into the first time, the second to fifth time, or more than the fifth time. Occupations were consolidated into the categories of clinicians, academics, or clinical tutors. A senior doctor was considered to be any clinician working at the consultant grade, while junior doctors were defined as interns, residents, registrars, or fellows. General practitioners, paediatricians, and general internal medicine physicians were all regarded as nonspecialists (‘generic’).

### Statistical analysis

Descriptive statistics were employed to compare the mean marks awarded for each of the 3 domains across all assessed students in relation to the examiner characteristics described above. The statistical significance of differences in mean scores was calculated using the 2-tailed independent t-test and analysis of variance as appropriate, with P-values > 0.05 considered to indicate statistical significance. The analysis was performed using IBM SPSS ver. 24.0 (IBM Corp., Armonk, NY, USA).

## Results

### Examiner characteristics

There were 517 students examined by 237 examiners across the OSCEs delivered in 2014 and 2016, producing a total of 1,536 domain marks for the final analysis. The examiner characteristics are presented in Table 1. Of the examiners, 132 (55.7%) were male, 225 (94.9%) were clinicians, and 130 (54.9%) were junior doctors. Furthermore, 129 of the respondents (54.4%) classified themselves as non-specialists (‘generic’ in Table 1), and 98 (41.1%) of the examiners had only evaluated 1 OSCE prior to participating in the present study.

### Examiner gender, occupation, and speciality

The examiner’s gender and occupation (academic, clinician, or clinical tutor) did not significantly impact domain score results (P> 0.05 for all comparisons) (Tables 2, 3). The scores of examiners who were clinicians were likewise not significantly influenced by their speciality (P> 0.05) (Table 4).

### Examiner seniority

Junior doctors scored consistently higher than senior doctors in all domains of OSCE assessment (Table 5). The difference in scoring was significant for generic communication (mean difference, 0.163; P= 0.01; 95% confidence interval [CI], 0.039 to 0.287) and clinical communication (mean difference, 0.177; P= 0.004; 95% CI, 0.058 to 0.295) by seniority.

### Experience in assessing

Examiner experience significantly impacted generic communication scores. Examiners who had assessed OSCEs more than 5 times previously awarded 0.14 (P= 0.037; 95% CI, 0.009 to 0.271) lower mark on average than examiners who were administering an OSCE for the first time, and 0.21 (P=0.023; 95% CI, 0.030 to 0.390) mark lower than those who had done so only 2–5 times (Table 6). Differences in clinical communication and physical examination domains scores were noted but were not statistically significant (P> 0.05 for all comparisons). Raw data are available from Supplement 1.

## Discussion

The assessment of communication performance is susceptible to significant bias associated with examiner experience and clinical seniority. Examiner gender, occupation, and speciality only produced trivial differences in the mean domain scores. The domain of physical examination was not significantly affected by any examiner characteristics in the present study.

### Physical examination domain marking

We propose that examination skills may be less susceptible to examiner bias because the process of patient physical examination is well-documented, fairly prescriptive, and widely accepted [8]. Thus, there is often little room for interpretation of the ‘correct’ way of executing this skill, affording greater objectivity in marking an examinee’s performance. In addition, physical examination technique can be improved upon with practice, but ultimately has a ceiling of achievement [9]. Thus, the differences in physical examination skills between OSCE candidates and examiners of varying seniority can be small, further minimising the potential for bias.

### Generic and clinical communication domains marking

Effective communication involves establishing a good interpersonal relationship to facilitate the exchange of information, as well as including patients in the decision-making process. The inherent complexity of this task implies that continuous, life-long refinement of this skill is possible, with no ‘ceiling of learning’ [9], as may be present for the skill of physical examination. Therefore, because senior clinicians have a greater richness of clinical experience, they may also have a better awareness of the subtleties of effective communication than their junior counterparts. This may explain the statistically significant, but small, biases for senior clinicians to mark examinees more harshly in the generic and clinical communication skills domains. Furthermore, communication skills are reported to be closely bound to self-concept, self-esteem, and personal style, and may be further affected by examiner bias as a result [9]. In keeping with our results, Park et al. [10] reported that examiners who had greater academic experience, and therefore presumably higher exposure to administering examinations as part of their educational role, tended to give significantly lower OSCE marks than those awarded by parttime faculty. Similarly, other studies employing OSCE-type assessment models have found that clinical experience (i.e., seniority) did not necessarily imply consistency and fairness when awarding marks [2].

### Finding

Our finding of an inverse relationship between the number of times an examiner had administered an OSCE and leniency in marking generic communication skills may be explained by clinical experience, a concept that encompasses both its own biases and those inherent to experience in assessing. It is known that as examiners assess more students, they mentally amalgamate previous performances to produce a standard against which to judge future candidates [3]. However, this form of bias is not unique to examiners with experience in assessing. All clinicians with a teaching role informally assess the clinical skills of students while they are on placements, and more senior examiners often have greater experience in teaching, rendering them more susceptible to this bias [3]. In addition to this, examiners use themselves as a reference point for assessment marking. This may cause their ratings to become harsher as they become more senior. A richer clinical experience may engender a greater appreciation of the centrality of clinical skills in ensuring quality patient care, as well as a greater understanding of the importance of the OSCE as a summative, high-stakes assessment. More senior clinicians may therefore feel morally obliged to the medical and patient community to provide a stringent and accurate assessment of performance. This bias was reproduced in the comparison of clinical communication scores of first-time examiners with those who had administered an examination more than 5 times, although the significance of this finding was borderline at P= 0.053. We suggest that the tendency of firsttime examiners to be more junior clinicians, and thus more recently exposed to communication skills assessments in their undergraduate training, may account for this finding, perhaps due to their increased stringency when assessing this skill in others.

### Limitations

The limitations of this study mostly relate to the large number of examiners involved, many of whom only examined 6 students (i.e., 1 OSCE ‘session’). Had the overall number of data points been small, this limitation might have been significant. However, as the results demonstrated that differences in scoring were present for some examiner characteristics but not for others, our findings are unlikely to have been due to artefacts. Another limitation warranting consideration is that unmeasured differences between examiners and students may have existed and impacted our results; however, these could not be analysed due to the large numbers present in these 2 groups. A remedy for this limitation may involve a controlled trial undertaken with a smaller number of examiners and examinees, or by using a standardised observed OSCE (e.g., a video) across all examiners. Future research may utilise such methodologies to add further rigour to findings in this particular field.

### Conclusion

In conclusion, our findings demonstrated small but statistically significant differences in the marks awarded for the communication aspects of undergraduate medicine OSCEs according to examining experience and clinician seniority. This effect did not persist when we analysed the marks awarded for the physical examination assessment domain. We believe that our data highlight the need for specific strategies to encourage more objective marking by examiners. We recommend training that outlines the marking criteria, teaching curriculum, and expected level of student performance in communication and examination skills as a strategy to reduce bias in OSCE assessment. This would allow examiners to mark students in a way that reflects their true performance, irrespective of examiner seniority or experience with assessment.

## Figures and Tables

**Table 1.** Examiner characteristics (n=237)

Characteristic	No. (%)
Gender	
Male	132 (55. 7)
Female	94 (39.7)
Unknown	11 (4.6)
Examining experience	
1st time examining	98 (41.4)
2nd–5th time examining	45 (19.0)
> 5 times examining	79 (33.3)
Unknown	15 (6.3)
Examiner occupation	
Clinician	225 (94.9)
Academic	158 (66. 7)
Clinical tutor	75 (31.6)
Unknown/unanswered	10 (4.2)
Examiner seniority	
Junior	130 (54.9)
Senior (GP/senior)	93 (32.9)
Unknown	14 (5.9)
Examiner specialty	
Generic (GP, paediatrics, medicine)	129 (54.4)
Specialised	51 (21.5)
Unknown/unanswered	57 (24.1)

GP, general practitioner.

**Table 2.** Scores by domain and by gender

	Gender	Mean	95% confidence interval
Clinical communication	M	7.27	7.23–7.31
	F	7.29	7.25–7.34
Generic communication	M	7.40	7.36–7.44
	F	7.38	7.34–7.43
Physical examination	M	7.11	7.06–7.16
	F	7.11	7.05–7.18

M, male; F, female.

**Table 3.** Scores by domain and by occupation

Domain	Occupation	Mean	95% confidence interval
Clinical communication	Clinician	7.32	7.24–7.39
	Academic	7.24	7.19–7.29
	Tutor	7.32	7.27–7.37
Generic communication	Clinician	7.42	7.35–7.50
	Academic	7.34	7.31–7.39
	Tutor	7.44	7.38–7.49
Physical examination	Clinician	7.15	7.05–7.24
	Academic	7.08	7.02–7.14
	Tutor	7.14	7.07–7.20

**Table 4.** Scores by domain and by specialty

Domain	Generic	Specialised	P-value
Generic communication	7.41	7.35	0.417
Clinical communication	7.28	7.28	0.956
Physical examination	7.11	7.20	0.345

**Table 5.** Influence of examiner seniority on the mean difference in domain scoring, with significance calculated using the 2-tailed t-test

Objective structured clinical examination domain	Junior	Senior	Significance	Mean difference	95% confidence interval
Generic communication	7.47	7.31	0.011	0.16	0.04 to 0.29
Clinical communication	7.37	7.19	0.003	0.18	0.06 to 0.29
Physical examination	7.16	7.09	0.348	0.07	-0.08 to 0.22

**Table 6.** Influence of examiner experience on mean domain scoring, with significance calculated by analysis of variance

Examiner experience	1st time (n = 98)	2nd to 5th time (n = 45)	> 5 times (n = 79)	P-value
Generic communication	7.44 ± 0.44	7.51 ± 0.56	7.30 ± 0.44	0.036
Clinical communication	7.34 ± 0.42	7.34 ± 0.51	7.21 ± 0.45	0.122
Physical examination	7.15 ± 0.53	7.13 ± 0.59	7.10 ± 0.58	0.875

Values are presented as mean±standard deviation.

## Supplementary material

Supplement 1. Data files are available from https://doi.org/10.7910/DVN/PBNE4K.

Supplement 2. Audio recording of the abstract.
